# Identification and Characterization of *Diaporthe citri* as the Causal Agent of Melanose in Lemon in China

**DOI:** 10.3390/plants14121771

**Published:** 2025-06-10

**Authors:** Yang Zhou, Liangfen Yin, Wei Han, Chingchai Chaisiri, Xiangyu Liu, Xiaofeng Yue, Qi Zhang, Chaoxi Luo, Peiwu Li

**Affiliations:** 1 Oil Crops Research Institute, Chinese Academy of Agricultural Sciences, Wuhan 430062, China; zhouyang01@caac.cn (Y.Z.); hw2695001057@126.com (W.H.); yuexf2017@caas.cn (X.Y.); zhangqi01@caas.cn (Q.Z.); 2 Key Lab of Horticultural Plant Biology, Ministry of Education, Huazhong Agricultural University, Wuhan 430070, China; yh@mail.hzau.edu.cn (L.Y.); chaisiri.ch@gmail.com (C.C.); xiangyuliu@webmail.hzau.edu.cn (X.L.); 3 Hubei Hongshan Laboratory, Wuhan 430070, China; 4 Experimental Teaching Center of Crop Science, College of Plant Science and Technology, Huazhong Agricultural University, Wuhan 430070, China; 5 College of Plant Science and Technology, Huazhong Agricultural University, Wuhan 430070, China; 6 Office of Agricultural Regulation, Department of Agriculture, Bangkok 10900, Thailand

**Keywords:** *Citrus limon*, melanose disease, *Diaporthe citri*, molecular identification, pathogenicity test

## Abstract

Lemon, widely used in food, medicine, cosmetics, and other industries, has considerable value as a commodity and horticultural product. Previous research has shown that the fungus *Diaporthe citri* infects several citrus species, including mandarin, lemon, sweet orange, pomelo, and grapefruit, in China. Although *D. citri* has been reported to cause melanose disease in lemons in China, key pathological evidence, such as Koch’s postulates fulfillment on lemon fruits and detailed morphological characterization, is still lacking. In May 2018, fruits, leaves, and twigs were observed to be infected with melanose disease in lemon orchards in Chongqing municipality in China. The symptoms appeared as small black discrete spots on the surface of fruits, leaves, and twigs without obvious prominent and convex pustules. *D. citri* was isolated consistently from symptomatic organs and identified provisionally based on the morphological characteristics. The identification was confirmed using sequencing and multigene phylogenetic analysis of ITS, *TUB*, *TEF*, *HIS*, and *CAL* regions. Pathogenicity tests were performed using a conidium suspension, and melanose symptoms similar to those observed in the field were reproduced. To our knowledge, this study provides the first comprehensive evidence for *D. citri* as a causal agent of melanose disease in lemons in China, including morphological characterization and pathogenicity assays on lemon fruits. This report broadens the spectrum of hosts of *D. citri* in China and provides useful information for the management of melanose in lemons.

## 1. Introduction

Lemon (*Citrus limon* (L.) Burm. F.) is rich in various bioactive compounds such as flavonoids and vitamin C, possessing both health-promoting and therapeutic potential [1]. As an important natural flavoring agent, food ingredient, and industrial raw material, it is widely used in food, pharmaceutical, and cosmetic industries [1,2]. Given these versatile applications, lemons are cultivated in over 100 countries and regions worldwide [3]. According to statistics, the global lemon cultivation area reached 1.08 million hectares, yielding 17.21 million metric tons in 2017 [4]. In 2019, China’s production reached 2.71 million metric tons, accounting for 10.7% of global output, with its 130,000-hectare cultivation area representing 13.5% of the world’s total—ranking third in both production volume and cultivation area rankings [5]. The southwestern provinces of Sichuan, Yunnan, and Chongqing constitute China’s primary lemon-producing regions, where lemon cultivation has become a significant source of income for local farmers [5].

Common lemon diseases include melanose (*Diaporthe citri*, anamorph *Phomopsis citri*), Phytophthora gummosis and fruit brown rot (*Phytophthora* spp.), bot gummosis (*Neofusicoccum parvum* and other *Botryosphaeriaceae*), citrus canker (*Xanthomonas citri* pv. *citri*), septoria spot (*Septoria citri*), sooty mold (*Capnodium* spp., associated with aphids), gray mold (*Botrytis cinerea*), penicillium fruit rot (*Penicillium digitatum* and *P. italicum*), scab (*Elsinoe* spp.), black spot (*Phyllosticta citircarpa*), anthracnose (*Colletotrichum* spp.), mal secco (*Plenodomus tracheiphilus*), and stem-end rot (*Lasiodiplodia theobromae*) [6,7,8,9,10,11,12,13]. Melanose caused by *D. citri* is distributed across major citrus-growing regions worldwide, including Asia (e.g., China), the Americas (e.g., United States), Africa (e.g., Mauritius), Oceania (e.g., Australia), and Europe (e.g., Portugal); all citrus species are susceptible, particularly grapefruits [14,15,16,17,18]. *D. citri* primarily infects leaves and fruits, with secondary stem-end rot development, and acts as a saprophyte on dead twigs [6,16]. This fungal disease exhibits a spectrum of symptoms ranging from spot-like lesions to characteristic tear-drop, mud-cake, and star-shaped patterns [6,14,19]. The pathogen’s variable symptomatology poses challenges for field diagnosis. The melanose disease causes a decline in fruit quality, which lowers the value of fruits during marketing and exportation [18]. Fruit appearance is a critical factor in determining commercial value; affected fruits are typically downgraded to juice processing due to cosmetic damage.

The *Diaporthe* genus fungi are well known as saprobic-, endophytic-, and pathogenic-plant parasites on economically significant plant cultivars [20,21]. Accurate identification of *Diaporthe* species is crucial for disease control and quarantine strategy development [22,23]. Molecular phylogenetic analysis using multi-locus DNA sequences has significantly improved the identification accuracy of *Diaporthe* species [16,20,24,25]. Particularly, combined analysis of the translation elongation factor 1-α gene (*TEF*), β-tubulin gene (*TUB*), calmodulin gene (*CAL*), and histone-3 gene (*HIS*) provides optimal resolution for *Diaporthe* species discrimination, facilitating pathogen tracking [24,25,26].

In November 2018, melanose disease was observed on lemons (cv. Eureka) in a 1.2-hectare orchard in the Chongqing municipality. The symptoms appeared as small black discrete spots (0.3 to 1 mm in size) on the surface of fruits, leaves, and twigs without obvious prominent and convex pustules. The disease incidence on fruits was estimated at approximately 3% based on counting diseased fruits on five randomly selected trees (>50 m apart). Chongqing municipality is a major lemon-producing region in China, with ‘Eureka’ being one of the predominant cultivated varieties and an important economic crop in the area. Currently, melanose has become one of the primary diseases affecting lemons, significantly reducing the commercial value of the fruits and causing substantial economic losses to growers.

Current research indicates that *D. citri* in China can infect various citrus species, including mandarins, sweet oranges, pomelos, and grapefruits [14,16,20]. During 2023, a comprehensive survey by Professor Hong-Ye Li across China’s major citrus-growing regions yielded 1287 *Diaporthe* isolates obtained from symptomatic plants displaying melanose (leaves/fruits), dieback, necrotic lesions with gummosis, cankers, or wood rot. Multi-locus phylogenetic analysis (combined with morphological assessment) resolved these isolates into 36 *Diaporthe* species, including 32 known species (14 first reported on citrus hosts; 2 new records for China) and 4 novel taxa: *Diaporthe gammata*, *D. jishouensis*, *D. ruiliensis*, and *D. sexualispora*. Focusing on *D. citri*, two representative strains were characterized: ZJUE-0254, isolated from gummosis-infected branches of *Citrus* hybrid ‘Hongmeiren’ (Changxing Island, Shanghai); ZJUE-0413, obtained from melanose lesions on fruits of *C. unshiu* (Shaoyang, Hunan Province). Pathogenicity assays included: In vitro inoculation on detached healthy twigs of *C. paradisi* and *C. reticulata* ‘Ponkan’; In vivo stem inoculation on young branches of *C. limon*, Citrus hybrid ‘Cocktail grapefruit’, and *Fortunella margarita*; Leaf inoculation on *C. tangerina* ‘Hongju’, *C. limon*, and *C. sinensis*. Both strains induced consistent symptoms: branch gummosis and leaf melanose, fulfilling Koch’s postulates [27]. However, previous studies exhibited three critical limitations: (1) an absence of morphological characterization for lemon-derived *D. citri* strains; (2) the failure to perform Koch’s postulates verification using lemon-isolated strains through original-host reinoculation; and (3) a lack of pathogenicity tests on lemon fruits, despite the substantial economic impact of melanose on fruit marketability.

This study aims to establish the first etiological system for lemon melanose in China by integrating morphological characterization, multi-locus phylogenetic analyses (ITS, TUB, TEF, CAL, HIS), and fulfillment of Koch’s postulates. The results will elucidate the causative agents and support targeted disease management strategies.

## 2. Material and Methods

### 2.1. Sample Collection and Disease Investigation

In November 2018, melanose outbreaks were investigated in a commercial ‘Eureka’ lemon orchard located in Tongnan District, Chongqing, China. Symptomatic tissues (fruits/leaves/twigs) were aseptically collected using surface-sterilized tools, immediately placed in sterile bags with ice packs, and transported to the laboratory [20]. Disease incidence was quantified via systematic S-pattern sampling, examining five randomly selected trees (>50 m spacing) [28].

### 2.2. Isolation and Morphological Characterization

Isolation. Pathogens were isolated using the tissue separation method. Diseased fruits, leaves, and twigs (five each) were collected, and severely infected tissues were cut into 0.5 cm × 0.5 cm segments. The segments were surface-disinfected with 75% ethanol (1 min) and 1% sodium hypochlorite (30 s) and rinsed three times with sterile water. Subsequently, they were transferred to potato dextrose agar (PDA) plates (200 g/L potato, 20 g/L glucose, 15 g/L agar) and incubated at 25 °C in complete darkness [29]. After mycelial growth emerged, hyphal tips were subcultured onto fresh PDA plates until sporulation. Single spores were isolated using a specialized microscope for single-spore isolation (Wuhan Heipu Science and Technology Ltd., Wuhan, China). Two spores per sample were picked from water agar plates with a glass needle and transferred to PDA, followed by incubation at 25 °C for 4 days in darkness.

Morphological Characterization. Colony characteristics (including color, texture, and size) were documented daily. Culture plate images were captured using a Canon EOS 600D digital camera (Canon Inc., Tokyo, Japan). Additionally, pycnidial morphology was examined with an OLYMPUS SZX16 stereomicroscope (Olympus Corporation, Tokyo, Japan). Furthermore, microscopic morphology (conidia, conidiophores, spore germination, and germ tubes) was analyzed under a bright-field microscope (Nikon DS-Ri2, Tokyo, Japan) at 400× magnification [14]. Conidial dimensions (length × width) were measured for 100 randomly (Nikon DS-Ri2, Tokyo, Japan) selected spores using a calibrated ocular micrometer. Colony and pycnidial colors were determined by comparison with a standard color chart according to Rayner’s method [26].

### 2.3. Pathogenicity Test

Preserved strains were reactivated on a PDA medium and cultured at 25 °C in darkness until sporulation. Pycnidia-derived conidia were aseptically transferred to sterile water using sterilized toothpicks, and the suspension was adjusted to 10^6^ conidia/mL for pathogenicity tests [26]. In December 2018, the pathogenicity of the isolate CQTN-1 was evaluated on lemon (7-year-old cv. Eureka) in the growth room. Drops of 300 µL conidia suspension with a concentration of 10^6^ conidia/mL (α and β spores) on cotton were inoculated on the surface of five healthy fruits, fixed with scotch tape, and then wrapped in a plastic bag (a cotton ball with water was placed in the plastic bag) to maintain wetness for 3 days. After that, the inoculated plants were placed in a growth room with 95% relative humidity and incubated under the condition of 12/12 h light/dark at 25 °C. Five healthy fruits treated with water were used as the control. Inoculation of leaves and twigs was performed using an in vitro inoculation method [30]. Young leaves and tender twigs from 7-year-old Eureka lemon (Citrus limon cv. Eureka) were surface-sterilized through sequential immersion in 1% NaClO (1 min) and ddH_2_O (1 min), then air-dried under sterile conditions. A 100 μL aliquot of conidial suspension (10^6^ spores/mL, α and β spores) was spot-inoculated on adaxial leaf surfaces and twigs. After air-drying, samples were transferred to 150 mm Petri dishes containing moistened sterile cotton balls and incubated at 25 °C in darkness. Disease progression was assessed daily until symptom development. Two biological replicates were included. To verify the identity of the pathogenic agent, tissue sections taken from the edges of lesions on fruits and leaves that had been experimentally infected and had shown symptoms were cultured on a PDA medium to re-isolate the fungus. The identity of the isolated strain was confirmed through molecular analysis using sequences from the ITS, *TUB*, *TEF*, *HIS*, and *CAL* regions, as detailed below [24,25].

### 2.4. Molecular Identification and Phylogenetic Analysis

Mycelial agar blocks (5-day-old cultures on PDA at 25 °C) were transferred to potato dextrose broth (PDB, 200 g/L potato, 20 g/L glucose) and incubated for 4 days under identical conditions. Genomic DNA was extracted from freeze-dried mycelium using an optimized SDS protocol, with DNA pellets resuspended in 30 μL nuclease-free water and stored at −20 °C. For phylogenetic reconstruction, 45 isolates were analyzed using sequences of ITS rDNA, *TUB*, *TEF*, *CAL*, and *HIS* loci. Amplification was performed in 50 μL reactions with 2× Taq Master Mix (Vazyme, Nanjing, China) under the following conditions: 94 °C for 5 min; 35 cycles of 94 °C/30 s, (ITS: 52 °C, *TUB*: 55 °C, *TEF*: 58 °C, *CAL*: 53 °C and *HIS*: 56 °C)/30 s, 72 °C/1 min; final extension at 72 °C for 10 min [12]. The sequences of ITS rDNA, *TUB*, *TEF*, *CAL*, and *HIS* locus of the 15 selected isolates were amplified as described previously [12], then sequenced, and 3 selected isolates (CQTN-1, CQTN-2, and CQTN-3) were deposited in GenBank (ITS: MZ701845 to MZ701847; *TUB*: MZ703264 to MZ703266; *TEF*: MZ703267 to MZ703269; *CAL*: MZ703270 to MZ703272; and *HIS*: MZ703273 to MZ703275). The phylogenetic tree was constructed using concatenated sequences of ITS-*TUB*-*TEF*-*HIS*-*CAL* (Table 1). Every character had equal weight, and gaps were handled as missing data. Maximum likelihood (ML) analysis was performed with MEGA 5.0 software under the K2+G model with uniform substitution rates.

A bootstrap analysis with 1000 replicates was conducted to evaluate clade reliability. Only branches with ML bootstrap support values (BS) greater than 50% were taken into consideration for ML phylogenetic inference; the program calculated the MLBS to evaluate the robustness of MLBS studies.

## 3. Results

### 3.1. Morphological Characterization of D. citri

**Figure 1:**
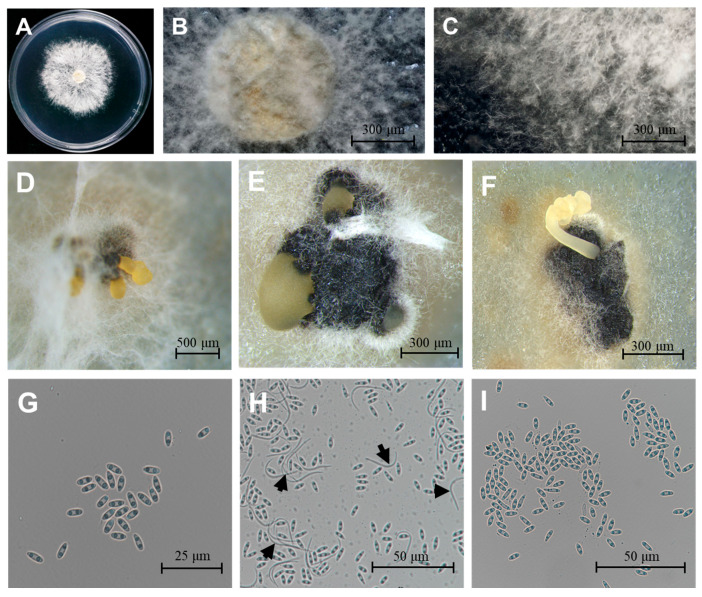
Morphological characteristics of *D. citri*. (**A**–**C**) Colony morphology on PDA medium after 5-day incubation at 25 °C. (**D**–**F**) Pycnidial conidiomata produced on PDA. (**G**,**I**) Ellipsoidal α-conidia. (**H**) Both α- and β-conidia (black arrows indicate β-conidia). Scale bars: 20 μm (**G**), 100 μm (**H**,**I**), 300 μm (**B**,**C**,**E**,**F**), 500 μm (**D**).

Using the tissue separation method and single-spore isolation method, 45 *D. citri* isolates were obtained. Three representative isolates (CQTN-1/2/3) were selected. The three isolates on PDA had colony diameters reaching between 55 mm and 71 mm and averaging 61 mm (Figure 1A). The colonies were overall circular, white to grayish-white, fluffy, and velvety, with smooth edges, and the mycelium radiated outwards from the inoculation point (Figure 1A–C). These characteristics corresponded to those of *D. citri* described previously [26]. Conidiomata were solitary or aggregated, black, spherical to globose (100 to 400 µm diam, Figure 1D–F). Conidial masses were yellowish and exuded from central ostioles. Two conidial morphotypes were observed, α-conidia: (6.62 ± 0.32 × 2.33 ± 0.11 μm; n = 100), aseptate, bi-guttulate, hyaline, and ellipsoid, length/width ratio = 2.84 (Figure 1G,I); β-conidia: (24.79 ± 1.25 × 1.31 ± 0.05 μm; n = 100), worm-shaped, hyaline, aseptate, slightly curved to spindle-shaped (Figure 1H, black arrows).

### 3.2. Phylogenetic Analysis

**Figure 2:**
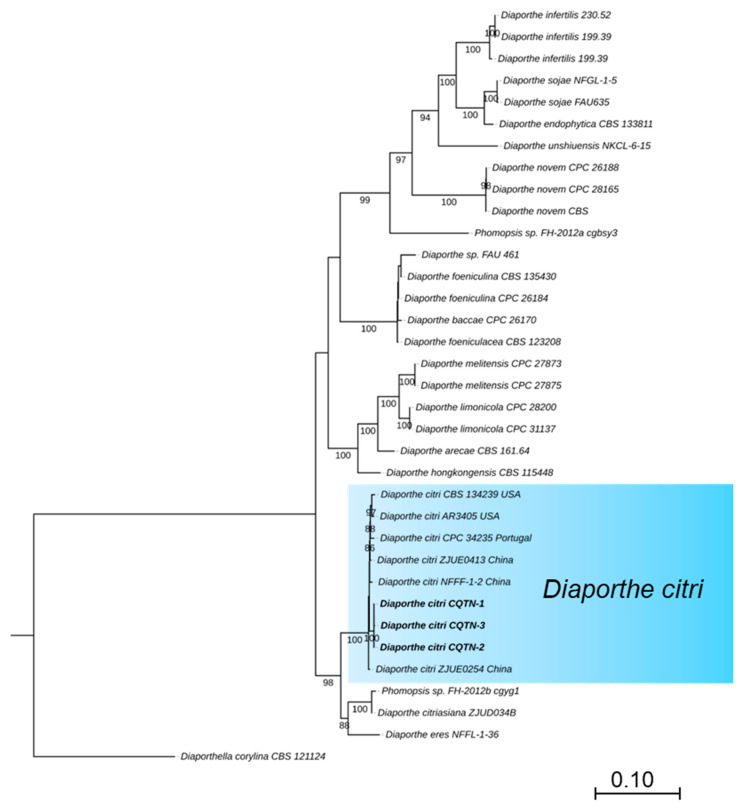
Phylogenetic tree of the *Diaporthe* species based on concatenated ITS, *TUB*, *TEF*, *HIS*, and *CAL* sequences. The majority rule consensus tree from maximum likelihood (ML) analysis was shown for the phylogenetic relationships of *Diaporthe* species, the tested isolates (CQTN-1, CQTN-2, and CQTN-3) grouped with *D. citri* strain CBS134239, AR3405, ZJUE0413, ZJUE0254, and CPC34235 in the same clade. The tree was rooted with *Diaporthella corylina* (CBS 121124). ML bootstrap values > 50% are shown at the branch nodes. Culture collection numbers are indicated behind the species names. Phylogram was generated with ML analysis based on the K2+G model.

For the preliminary identification, the MegaBlast search was performed for the ITS region of three isolates in NCBI’s GenBank nucleotide database (Table 1). BLAST analysis (MEGA version 11) indicated that the amplified ITS sequences were identical and showed the highest identity of 99% with that of *D. citri* (MZ224574, base pairs matching 565/567 MN816401, and MN816398), while *TUB*, *TEF*, *CAL*, and *HIS* sequences showed 100% identity with that of *D. citri* (*TUB*: KC357427, base pairs matching 501/501, KC357458 and KC357453; *TEF*: JQ954673, base pairs matching 327/327, MW221501 and KC357515; *CAL*: MW221703, base pairs matching 533/533 and KC357466; *HIS*: MW221596, base pairs matching 475/475 and MW221597). The phylogenetic tree of *Diaporthe* species was based on concatenated that the tested isolates grouped in the same clade with *D. citri* strains ZJUE0413 (China), NFFF-1-2 (China), CPC 34235 (Portugal), CBS134239 and AR3405 (USA) (Figure 2). The phylogenetic tree of the *Diaporthe* species was based on concatenated ITS, *TUB*, *TEF*, *HIS*, and *CAL* sequences. The majority rule consensus tree from maximum likelihood (ML) analysis was shown for the phylogenetic relationships of *Diaporthe* species, the tested isolates (CQTN-1, CQTN-2, and CQTN-3) grouped with *D. citri* strain CBS134239, AR3405, CPC34235, ZJUE0413, ZJUE0254, and NFFF-1-2 in the same clade. The phylogenetic analysis from the combined dataset of ITS, *TUB*, *TEF*, *HIS*, and *CAL* was highly effective and strongly supported grouping together, as shown by the 100% MLBS. The tree was rooted with *Diaporthella corylina* (CBS 121124) as an outgroup. ML bootstrap values >50% are shown at the branch nodes. Culture collection numbers are indicated behind the species names. A phylogram was generated with ML analysis based on the K2+G model (Figure 2).

### 3.3. Pathogenicity Test Results

**Figure 3:**
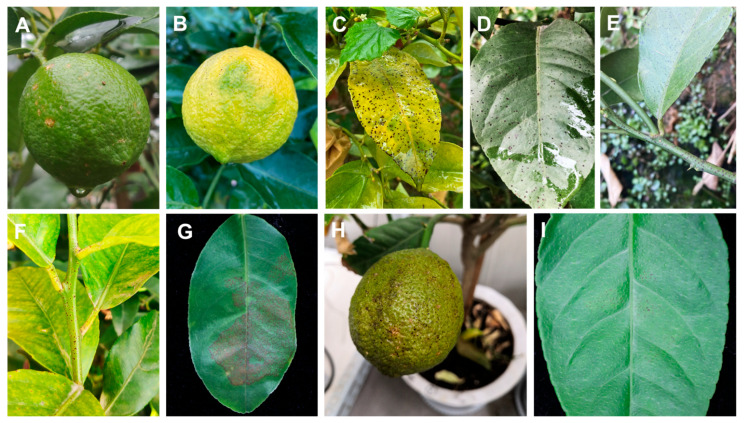
Symptomatology and pathogenicity assessment of citrus melanose caused by *D. citri*. (**A**,**B**) Field symptoms on fruits with characteristic melanized punctate lesions (0.3–1 mm diam). (**C**,**D**) Leaf lesions under natural infection. (**E**,**F**) Twigs symptoms observed in orchard conditions. (**G**) Artificially inoculated detached leaves showing enhanced lesion density due to controlled spore concentration (1 × 10^6^ conidia/mL). (**H**) Potted plant inoculation reproducing field-identical fruit symptoms. (**I**) Detached leaf assay results.

Melanose disease was observed on fruits, twigs, and leaves, appearing as slightly sunken black or dark brown water-soaked spots at the infection sites, often surrounded by yellow halos. Some lesions ruptured, exuding dark brown to black gummy substances, which gradually harden and protrude, resembling adhered fine sand particles, forming “tear-stain” or “muddy” lesions. Field observations showed that the lesions typically appeared as melanized punctate spots (0.3–1 mm in diameter) on citrus surfaces (Figure 3A–F), showing 3% disease incidence in sampled orchards. In pathogenicity tests, healthy fruits on potted lemon plants as well as detached leaves and twigs were used to test the pathogenicity of the strains. Three weeks after inoculation, discrete spots with black color appeared on the surface of the five inoculated lemon fruits in each of the independent experiments. Symptoms were similar to those observed in the field (Figure 3H). The leaves and twigs were inoculated using an in vitro inoculation method. Four weeks after inoculation, black dense spots were observed on the surface of the leaves, and the symptoms were consistent with those in the field (Figure 3G,I). Due to the high concentration of conidia in the suspension used for inoculation, the density of spots on leaves was greater than that in the field (Figure 3G). After in vitro inoculation, no disease spots were found on the twigs. Similarly, no symptoms were observed on control fruits, leaves, and twigs. Re-isolation was performed from symptomatic, artificially inoculated fruits and leaves following Koch’s postulates. *D. citri* was re-isolated from the inoculated fruits and leaves with a re-isolation frequency of 100%.

## 4. Discussion

This study provides the first comprehensive evidence establishing *Diaporthe citri* as a causal agent of melanose disease on lemon (*Citrus limon*) in China, fulfilling Koch’s postulates with lemon-isolated strains (CQTN-01 to CQTN-03) and significantly expanding the known host range of this economically important pathogen. While *D. citri* has been reported on lemon previously (Xiao et al., 2023) [27], our work is the first to: (1) demonstrate full pathogenicity through reinoculation of lemon-derived strains onto lemon plants; (2) focus specifically on fruit infections—the most commercially damaging phase—which were overlooked in earlier studies; and (3) provide detailed morphological characterization of lemon-associated *D. citri*, including conidiomata, α/β conidia, and cultural characteristics. Our findings align with prior reports of *D. citri*’s broad host adaptability across citrus species [6,27] but crucially advance the field by addressing gaps in strain specificity, disease impact, and morphological documentation.

Morphological characterization remains a key tool for fungal identification. Here, *D. citri* formed grayish-white, circular colonies on PDA medium with a fluffy to velvety texture, smooth margins, and radially expanding mycelium from the inoculation point-matching the typical description of *D. citri* [6,26]. Conidia constitute the primary inoculum source for citrus melanose and are critical for disease cycle progression [17,31]. We identified two conidial morphotypes: ellipsoidal α-conidia (6.62 × 2.33 μm) and filiform β-conidia. The observed α-conidial dimensions (6.62 × 2.33 μm) were marginally smaller than prior reports (7–9 × 2–3 μm), potentially attributable to intraspecific variation or culture conditions, as documented by Gomes for *Diaporthe* strains from diverse regions [20]. While *D. citri* can be provisionally identified through colony morphology, pycnidial conidiomata, and conidial characteristics, its morphological similarities to *D. passifloricola*, *D. durionigena*, *D. rosae*, and *D. ueckerae* necessitate additional diagnostic approaches for accurate species delineation [6,32,33,34].

Recent advances in *Diaporthe* population identification related to citrus hosts have been remarkable [24,25]. The application of multi-locus phylogenetic analyses has established a more precise taxonomic framework for these fungal pathogens, enabling accurate identification and classification of citrus-associated *Diaporthe* species, which provides crucial information for developing effective disease management strategies [16,25,26]. Comprehensive molecular identification using multi-locus sequence analysis (ITS, *TUB*, *TEF*, *HIS*, *CAL*) serves as reliable evidence for *Diaporthe* species determination [24]. The complete congruence (100% identity) of protein-coding genes (*TUB*, *TEF*, *CAL*, *HIS*) with reference strains, as opposed to the 99% sequence similarity observed in ITS regions, validates the conclusions of Udayanga regarding the superior discriminatory power of protein-coding genes for *Diaporthe* species demarcation [14,25]. Phylogenetic analysis grouped the Chongqing isolates into a clade comprising strains from China, Portugal, and the United States, providing strong support for the hypothesis of *D. citri* as a cosmopolitan pathogen exhibiting minimal geographical differentiation.

The verification of Koch’s postulates is an essential experimental procedure in pathogenicity studies [35]. In this study, artificial inoculation with conidial suspensions on fruits and leaves successfully fulfilled Koch’s postulates. Distinct black, dense spots were observed on the surface of inoculated fruits and leaves, exhibiting symptoms identical to natural field infections. However, symptom development was absent on inoculated twigs, indicating either tissue-specific susceptibility or an extended latent period for xylem colonization. This finding provides important guidance for field disease monitoring. In contrast to pathogens with rapid symptom development, *D. citri* demonstrates a significantly longer incubation period. Reliable inoculation requires strictly controlled environmental conditions, namely sufficient air humidity, appropriate temperature, optimal spore concentration, and targeted inoculation of young, susceptible host tissues.

Our research revealed a disease incidence rate of 3%, significantly lower than that reported for other citrus varieties [16]. Three hypotheses can explain this. First, the sampling site was a commercial orchard with routine fungicide applications and advanced disease prevention measures, reflecting high management standards. Second, citrus melanose disease may still be in the early epidemic phase in Chongqing. Third, the ‘Eureka’ lemon cultivar might exhibit inherent disease tolerance, though this requires further investigation. For improved disease control, we suggest enhancing field management through timely pruning to maintain optimal ventilation and meticulous removal of dead branches to eliminate primary inoculum sources. Priority should be given to evaluating the disease-resistance potential of different varieties and selecting the most appropriate disease-resistant varieties for local conditions. It is equally important to strengthen field disease monitoring systems and establish rapid detection methods to enable early detection and timely implementation of control measures. When required, fungicide applications ought to be considered to suppress the pathogen population and reduce the risk of citrus melanose outbreaks.

## 5. Conclusions

This study provides the first comprehensive evidence for *D. citri* as a causal agent of melanose disease in lemons in China, including morphological characterization and pathogenicity assays on lemon fruits. Through morphological characterization and multi-locus phylogenetic analysis (ITS, *TUB*, *TEF*, *CAL*, *HIS*), the pathogen was conclusively identified, fulfilling Koch’s postulates. The disease manifested as typical small black spots on lemon fruits and leaves, consistent with melanose symptoms observed on other citrus species. This finding expands the known host range of *D. citri* in China and provides critical insights for the management of melanose disease in lemons.

## Figures and Tables

**Table 1:** GenBank accession numbers of isolates used in this study.

*Diaporthe* Species	Isolate Number ^1,2^	GenBank Accession Numbers ^3^				
		ITS	*TUB*	*TEF*	*CAL*	*HIS1*
*D. citri*	NFFF-1-2	MN816394	MN894454	MN894415	MN894355	MN894380
*D. eres*	NFFL-1-36	MN816413	MN894473	MN894434	MN894365	MN894396
*D. sojae*	NFGL-1-5	MN816422	MN894482	MN894443	MN894371	MN894405
*D. unshiuensis*	NKCL-6-15	MN816430	MN894490	MN894451	MN894377	MN894412
*D. arecae*	**CBS 161.64** IT	KC343032	KC344000	KC343758	KC343274	KC343516
*D. baccae*	CPC 26170	MF418351	MF418510	MF418430	MF418185	MF418265
*D. citri*	**AR3405** T	KC843311	KC843187	KC843071	KC843157	MF418281
*D. citri*	**CBS 134.239** T	KC357553	KC357456	KC357522	KC357488	MF418280
*D. citri*	CBS 144227	MH063904	MH063916	MH063910	MH063892	MH063898
*D. citri*	ZJUE0254	ON035566	ON221775	ON049542	ON221724	ON113064
*D. citri*	ZJUE0413	OP218124	OP265643	OP265579	OP265451	OP265515
*D. citriasiana*	**CGMCC3.15224** T	JQ954645	KC357459	JQ954663	KC357491	MF418282
*D. citrichinensis*	**CGMCC3.15225** T	JQ954648	MF418524	JQ954666	KC357494	KJ490516
*D. citrichinensis*	ZJUD034B	KJ210539	KJ420829	KJ210562	KJ435042	KJ420879
*D. cytosporella*	**CBS 137.020** T	KC843307	KC843221	KC843116	KC843141	MF418283
*D. endophytica*	**CBS 133.811** T	KC343065	KC344033	KC343791	KC343307	KC343549
*D. foeniculina*	**CBS 123.208** T	KC343104	KC344072	KC343830	KC343346	KC343588
*D. foeniculina*	CBS 135430	KC843301	KC843215	KC843110	KC843135	MF418284
*D. foeniculina*	CPC 26184	MF418365	MF418525	MF418444	MF418199	MF418285
*D. hongkongensis*	**HKUCC 9104** T	KC343119	KC344087	KC343845	KC343361	KC343603
*D. infertilis*	**CBS 230.52** T	KC343052	KC344020	KC343778	KC343294	KC343536
*D. infertilis*	CBS 199.39	KC343051	KC344019	KC343777	KC343293	KC343535
*D. infertilis*	CPC 20322	KC343053	KC344021	KC343779	KC343295	KC343537
*D. limonicola*	**CBS 142.549** T	MF418422	MF418582	MF418501	MF418256	MF418342
*D. limonicola*	CPC 31137	MF418423	MF418583	MF418502	MF418257	MF418343
*D. melitensis*	**CBS 142.551** T	MF418424	MF418584	MF418503	MF418258	MF418344
*D. melitensis*	CPC 27875	MF418425	MF418585	MF418504	MF418259	MF418345
*D. novem*	**CBS 127.270** T	KC343156	KC344124	KC343882	KC343398	KC343640
*D. novem*	CPC 26188	MF418426	MF418586	MF418505	MF418260	MF418346
*D. novem*	CPC 28165	MF418427	MF418587	MF418506	MF418261	MF418347
*D. sojae*	**CBS 139.282** ET	KJ590719	KJ610875	KJ590762	KJ612116	KJ659208
*D* *iaporthella corylina*	**CBS 121124** T	KC343004	KC343972	KC343730	KC343246	KC343488

^1^ IT = ex-isotype, T = ex-type, and ET = ex-epitype cultures are indicated in isolate number with bold characters. ^2^ AR = Corresponding author’s personal collection of A.Y. Rossman; CBS = Westerdijk Fungal Biodiversity Institute (formerly CBSKNAW), Utrecht, The Netherlands; CGMCC = China General Microbiological Culture Collection, China; CPC = Culture collection of P.W. Crous, housed at Westerdijk Fungal Biodiversity Institute, Utrecht, The Netherlands; HKUCC = University of Hong Kong Culture Collection, Department of Ecology and Biodiversity, Hong Kong, China; and ZJUD = *Diaporthe* species culture collection at the Institute of Biotechnology, Zhejiang University, Hangzhou, China. ^3^ ITS = nuclear ribosomal internal transcribed spacer regions; *TEF* = translation elongation factor 1-α gene; *TUB* = beta-tubulin gene; *CAL* = calmodulin gene; and *HIS* = histone-3 gene.

## Data Availability

The original contributions presented in this study are included in the article. Further inquiries can be directed to the corresponding authors.
